# Editorial: Mechanisms of resistance and host responses to RNA virus infections

**DOI:** 10.3389/fpls.2026.1957527

**Published:** 2026-08-25

**Authors:** Sumit Jangra, Amalendu Ghosh, Govind Pratap Rao

**Affiliations:** 1 UF/IFAS Tropical Research and Education Center, Homestead, FL, United States; 2 Insect Vector Laboratory, Advanced Center for Plant Virology, Division of Plant Pathology, ICAR-Indian Agricultural Research Institute, New Delhi, India; 3 Advanced Center for Plant Virology, Division of Plant Pathology, ICAR-Indian Agricultural Research Institute, New Delhi, India

**Keywords:** plant RNA viruses, host-virus interactions, hormonal signaling, functional genomics, genome editing

## Introduction

Plant viral diseases are one of the major constraints to crop production and sustainable agriculture. Worldwide, nearly 47% of emerging and re-emerging plant diseases are caused by plant viruses (Jones, 2021). These viral diseases are reported to cause annual economic losses of US$60 billion (Wei et al., 2010). Recently, in 2025, the International Committee on Taxonomy of Viruses (ICTV) recognized 2,392 plant-infecting agents, including viruses, viroids, and satellite agents, and classified them into 247 genera, 55 families, and 25 orders (Rubino et al., 2025). This remarkable diversity among plant viruses is also reflected in the wide range of transmission mechanisms they have evolved to ensure their survival, spread, and successful infection in host plants. To comprehend this diversity, a plant virus transmission database was created, compiling 1,600 plant viruses, their mode of transmission, and vectors (https://library.wur.nl/WebQuery/virus) (Peters et al., 2024). Among the plant viruses, RNA viruses pose a particularly formidable challenge because their error-prone replication, high mutation rates, frequent recombination, and rapid evolution enable them to adapt swiftly to new hosts, evade plant immunity, and overcome genetic resistance.

## Overview of the Research Topic

Deciphering the molecular basis of host responses to RNA virus infection is therefore essential for strengthening crop protection and safeguarding global food security. Although many factors involved in viral replication and transmission have been identified, the precise pathways governing most host-virus systems remain unresolved. Over the past decade, advances in next-generation sequencing, including genomics, transcriptomics, proteomics, and metabolomics, as well as functional genomics approaches such as RNA interference and genome editing, have transformed the study of plant antiviral defence and accelerated the development of resistant cultivars (Samarskaya et al., 2025). This Research Topic was designed to advance our understanding of host plant–RNA virus interactions and translate emerging insights into more effective and sustainable crop protection strategies.

This Research Topic brings together 10 articles from 10 countries, including eight original research articles and two reviews. The contributions encompass a diverse range of crops, viral pathosystems, and experimental approaches, reflecting both the complexity of plant antiviral defence mechanisms and the broad scope of contemporary research in plant virology. Despite investigating distinct host–virus systems, the contributions converge on several overarching themes that enrich our understanding of antiviral resistance, reveal common principles governing plant–virus interactions, and highlight emerging opportunities for developing durable and sustainable disease management strategies.

## Discussion of the contributing articles

Several studies highlighted the complex molecular responses underlying RNA plant–virus interactions. Molad et al. associated tomato brown rugose fruit virus (ToBRFV)-induced leaf deformities with altered auxin and mitogen-activated protein kinase signalling, emphasizing the role of hormone-mediated pathways in symptom development. Nunna et al. demonstrated that co-infection by wheat streak mosaic virus and triticum mosaic virus extensively reprograms the wheat transcriptome, exacerbating disease through coordinated suppression of chloroplast-related genes. Similarly, Mutethia et al. identified distinct transcriptional signatures between symptomatic and symptomless shoots of rose rosette virus-infected plants. All these studies reveal that plant antiviral responses are driven through interconnected hormonal regulations, signaling networks, and broad transcriptional remodeling that determine disease development.

The second major theme is the discovery and functional validation of host genes that govern resistance or susceptibility to RNA viruses. Yang et al. characterized the *Remorin* gene family in sugarcane and implicated *ScREM1.5e* genes in the response to sugarcane mosaic virus, offering new insight into membrane-associated defense. Potts et al. identified members of the GYF gene family in *Vanilla planifolia* and examined their expression during Cymbidium mosaic virus infection, expanding the pool of candidate regulators of antiviral responses. From the opposite perspective, Polo-Oltra et al. investigated *ParPMC* as a susceptibility factor in plum pox virus infection and validated its function by silencing an ortholog in *Nicotiana benthamiana*. Collectively, these findings expand our understanding of the molecular basis of host–virus interactions and offer promising targets for developing durable virus-resistant crop varieties.

The Research Topic also demonstrates the transformative potential of modern biotechnology in developing virus-resistant crops. Le et al. enhanced papaya resistance to papaya ringspot virus through CRISPR/Cas9-mediated editing of the translation initiation factor eIF4E, whereas Zisi et al. demonstrated that a single amino acid substitution in ToBFRV can overcome resistance in a newly developed tomato cultivar. Together, these studies define both the promise and the limitations of resistance breeding, emphasizing that while genome editing offers powerful tools for crop improvement, the rapid evolution of RNA viruses necessitates durable, multilayered, and continuously monitored resistance strategies.

The review articles included in this topic place these experimental advances within a broader biological and translational framework. Roy et al. explore the dynamic coevolution of plant RNA viruses and their hosts, highlighting the complex molecular interactions that shape infection and adaptation. Shilpha and Kang summarize recent advances in viral resistance research in *Capsicum*, emphasizing progress in resistance genetics, functional genomics, and modern breeding approaches. Together, these reviews underscore the importance of integrating molecular biology, genomics, plant pathology, and crop breeding to develop sustainable and durable strategies for managing viral diseases.

## Conclusion

The studies presented in the Research Topic collectively demonstrate that resistance to RNA viruses is a dynamic and multifaceted trait governed by interconnected signaling pathways, regulatory networks, host susceptibility factors, and the continual coevolution of plants and viruses. Together, they provide new mechanistic insights into antiviral defence while showcasing the transformative potential of high-throughput sequencing, functional genomics, transcriptomics, and genome editing for accelerating the development of virus-resistant crops.

Looking ahead, achieving durable resistance will require integrating molecular biology, systems biology, precision phenotyping, computational approaches, and advanced breeding technologies to identify and deploy resistance mechanisms capable of withstanding the rapid evolution of RNA viruses. We hope this collection will inspire further interdisciplinary research, fosters a deeper understanding of plant–RNA virus interactions, and accelerates the translation of fundamental discoveries into sustainable strategies for protecting crops and strengthening global food security.
